# Lip Repositioning Surgery for Nonskeletal Excessive Gingival Display: A Clinical Case Report

**DOI:** 10.1155/crid/7738188

**Published:** 2026-07-29

**Authors:** Meiya Suo, Yu Huan, Chenghong Cao, Shuya Zhang, Lan A., Wenzhou Xu

**Affiliations:** ^1^ Stomatological Hospital, Jilin University, Changchun, China, jlu.edu.cn

**Keywords:** excessive gingival display, facial esthetics, gummy smile, lip repositioning surgery

## Abstract

Excessive gingival display, or gummy smile, caused by upper lip elevator muscle hyperactivity presents a significant esthetic challenge. This case report details the successful management of a 28‐year‐old female patient with an objectively measured dynamic gingival exposure of 4 mm. Preoperative evaluations confirmed normal clinical crown dimensions and healthy periodontal tissues, definitively ruling out altered passive eruption. The patient underwent conventional lip repositioning surgery (LRS) featuring a partial‐thickness mucosal excision and meticulous wound closure with 6‐0 polytetrafluoroethylene (PTFE) sutures to mechanically restrict upper lip mobility. The dynamic gingival display decreased from 4.0 mm preoperatively to 1.0 mm at 2 weeks and was stably maintained at 1.5 mm at the 1‐year follow‐up, meeting the treatment success criteria (≤ 2 mm). The procedure established a harmonious dentolabial relationship with high patient satisfaction and no complications, such as severe scarring, labial paresthesia, or relapse. These findings demonstrate that, based on accurate differential diagnosis and strict case selection, LRS serves as a safe, minimally invasive, and highly predictable intervention yielding excellent short‐to‐medium‐term esthetic outcomes for muscular hyperactive gummy smiles.

## 1. Introduction

In recent years, alongside the escalating focus on smile esthetics among both the general public and dental professionals, the diagnosis and management of the gummy smile have emerged as a focal point in clinical dentistry [1]. In the realm of smile esthetics, a gingival display of ≤ 2 mm is considered a fundamental criterion for an attractive smile; some authors consider a gummy smile to be present when the exposure exceeds 3 mm, representing a prevalent esthetic impairment [2, 3]. This condition is highly common in the general population, with a reported prevalence ranging from 10.5% to 29%. It exhibits a higher incidence in females, who also demonstrate approximately twice the level of esthetic concern regarding this condition compared with males [3, 4]. The etiology of this condition is multifactorial and includes skeletal discrepancies such as vertical maxillary excess (VME), periodontal variables like altered passive eruption (APE), and soft‐tissue factors including a short upper lip or hypermobility of the lip elevator muscles [5] Previous studies have indicated that upper lip elevator muscle hyperactivity is the most frequent etiology of the gummy smile [6]. Currently, various corrective techniques are employed for the management of this condition, including gingivectomy, orthognathic surgery, lip repositioning surgery (LRS), and botulinum toxin injections [7]. The selection of an appropriate treatment modality must be strictly dictated by the underlying pathogenic factors. Widely advocated as a safe, conservative, and minimally invasive procedure, LRS serves as an alternative treatment option. Despite its immediate postoperative success and high patient acceptance, the long‐term predictability of conventional LRS remains highly controversial in the current literature due to significant relapse rates driven by the persistent, powerful contraction forces of the perioral musculature [8]. To counteract these muscular forces and enhance the longevity of the surgical outcomes, several technical modifications have recently been evaluated. For instance, Adel introduced a dual‐layered suturing technique designed to provide internal soft tissue fixation; however, clinical trials indicated that although this approach could delay early relapse, complete recurrence of the gummy smile inevitably occurred by 6 months postoperatively [9]. Similarly, a more aggressive modification utilizing deliberate muscle fiber transection and mucosal scarring demonstrated an ability to maintain surgical correction for up to 6 months, but ultimately resulted in full relapse by 9 months. Consequently, adjunctive and repeated botulinum toxin injections are frequently recommended to manage muscular tension during the healing phase [10]. This article reports the case of a 28‐year‐old female patient with a gummy smile. By employing LRS, we effectively restricted the dynamic upward elevation of the upper lip and significantly improved smile esthetics. This report is aimed at providing a minimally invasive and highly effective clinical rationale and reference for the management of similar cases.

## 2. Case Presentation

**Figure 1:**
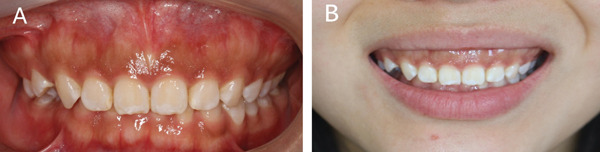
(A) Intraoral view showing normal gingival color, contour, and texture. (B) Dynamic view demonstrating a high smile line during a natural smile.

A 28‐year‐old female patient presented with a chief complaint of esthetic dissatisfaction due to excessive gingival display upon smiling. Her past medical and personal histories were unremarkable. Extraoral examination revealed general facial symmetry and a harmonious proportion of the lower third of the face. At rest, the patient′s upper lip length, measured from the base of the nose (subnasale) to the lower border of the upper lip at rest, was 20.5 mm. This value falls within the normal range for adult females (typically 18–22 mm). Intraoral examination indicated optimal oral hygiene. The gingiva exhibited normal color, contour, and texture. Full‐mouth periodontal probing depths (PPD) ranged from 1 to 3 mm with no clinical attachment loss (CAL). The average width of the attached gingiva was approximately 4 mm, and the width‐to‐length ratio of the maxillary anterior teeth marked by the T‐gauge was 75%–80%, which is consistent with the ideal clinical crown proportions. (Figure 1A). The clinical crown length of the maxillary right central incisor was measured as 10.15 mm from the incisal edge to the free gingival margin, which falls within the normal range (9.5–11.5 mm) for an adult female. Dynamic smile analysis revealed a high smile line during a natural smile. During a maximum smile, the excessive gingival display in the maxillary anterior region was objectively measured at 4 mm using a calibrated periodontal probe, spanning from the gingival margin to the lower border of the upper lip (Figure 1B). Observation of the upper lip kinematics during smiling demonstrated a pronounced upward elevation, indicating hypermobility and hypercontraction of the levator labii superioris (LLS) and levator labii superioris alaeque nasi (LLSAN) muscles.

Based on the aforementioned clinical findings and dynamic assessments, a diagnosis of muscular hyperactive excessive gingival display was established. The patient refused botulinum toxin due to its temporary effect, which would require repeated injections and incur extra time and expense. Therefore, LRS was chosen as a one‐time definitive intervention. Considering the definitive etiology of upper lip elevator muscle hyperactivity and the patient′s explicit request for a rapid therapeutic outcome, LRS was planned. The primary objective of this intervention was to mechanically restrict the dynamic upward elevation of the upper lip, thereby effectively masking the excessive gingival exposure during smiling.

### 2.1. Preoperative Preparation

Preoperative preparation included a comprehensive periodontal evaluation. Routine blood tests and coagulation profiles were confirmed to be within normal limits, effectively ruling out any surgical contraindications. The surgical procedure, along with potential intraoperative and postoperative complications, was thoroughly explained to the patient. Written informed consent was obtained from the patient for both the surgical intervention and the subsequent publication of this case report.

### 2.2. Surgical Procedure

**Figure 2:**
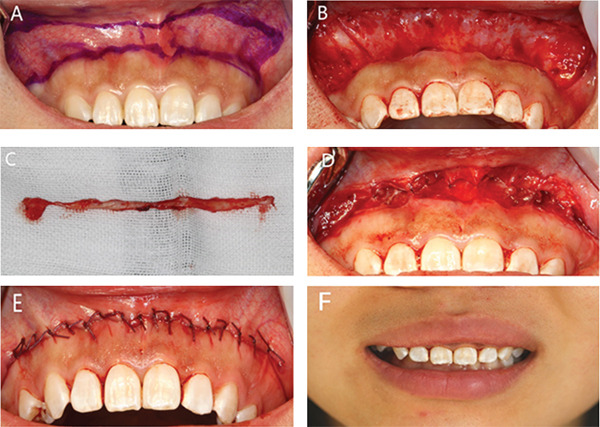
(A) Incision design. (B) Dissection and removal of the delineated tissue strip via a superficial partial‐thickness incision. (C) Excised tissue strip. (D) Elimination of minor dead spaces. (E) Closure of the exposed connective tissue borders using 6‐0 PTFE sutures. (F) Immediate postoperative view.

The procedure is as follows: (1) Following routine surgical preparation and draping, local infiltration anesthesia was administered. (2) A mucosal excision outline was marked in the upper labial vestibule (Figure 2A). Along this outline, a partial‐thickness horizontal incision was made 1 mm coronal to the mucogingival junction (MGJ). Subsequently, two parallel vertical incisions extending 10 mm apically were performed at both ends. (3) The apical endpoints of the vertical incisions were connected by a second horizontal incision, followed by the sharp dissection and removal of the delineated mucosal tissue strip (Figure 2B,C). (4) After eliminating minor dead spaces and achieving adequate undermining for tension release (Figure 2D), (5) the surgical wound was meticulously closed using 6‐0 polytetrafluoroethylene (PTFE) sutures (Figure 2E). No periodontal dressing was applied. This procedure effectively shortened the vestibular depth and relocated the upper lip muscle attachment to a more coronal position, thereby establishing a mechanical restriction against the excessive upward movement of the upper lip (Figure 2F).

### 2.3. Postoperative Instructions

Postoperative instructions included a routine prescription of oral antibiotics for 3–5 days, oral analgesics to be taken as needed for significant pain, and an antimicrobial mouthrinse (0.12% chlorhexidine gluconate, twice daily for 2 weeks). The patient was advised to apply extraoral ice packs intermittently for the first 24 h to minimize edema. Furthermore, she was strictly instructed to restrict exaggerated facial expressions, particularly wide smiling, for a duration of 2 weeks to prevent early suture displacement.

### 2.4. Criteria for Treatment Success and Follow‐up Schedule

The treatment success criteria were defined as a reduction in dynamic gingival display to ≤ 2 mm, maintenance of a harmonious smile line, absence of significant surgical complications (e.g., severe scarring or labial paresthesia), and high patient satisfaction. Scheduled follow‐up visits were established at 2 weeks and 1 year postoperative.

## 3. Result

**Figure 3:**
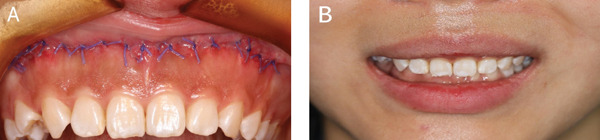
(A) Clinical view at 2 weeks postoperatively showing optimal wound healing with stable gingival architecture. (B) Decreased gingival exposure upon dynamic smiling.

At the 2‐week postoperative follow‐up for suture removal, the surgical site exhibited optimal healing. The wound was free of erythema, edema, and active bleeding. No ecchymosis or perioral swelling was noted, and the patient reported no discomfort. To quantify the immediate surgical effect, dynamic smile analysis was performed using a calibrated periodontal probe. The excessive gingival display during a maximum controlled smile was measured at exactly 1.0 mm, representing an initial overcorrection with a 3.0‐mm absolute reduction from the 4.0 mm preoperative baseline (Figure 3A,B).

**Figure 4:**
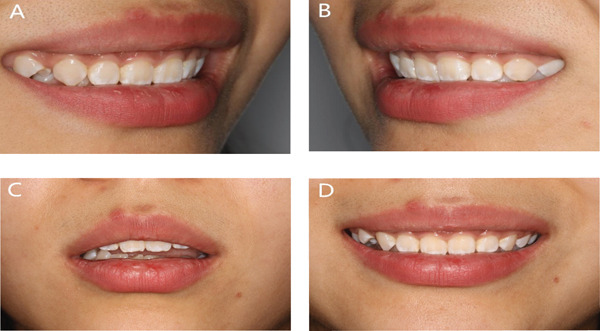
(A–D) Clinical views at the 1‐year postoperative follow‐up demonstrating highly satisfactory esthetic outcomes.

At the 1‐year follow‐up examination, comprehensive intraoral and extraoral photographs demonstrated stable gingival architecture. The gingival display upon smiling remained significantly reduced compared with the preoperative baseline. Objective remeasurement using the same calibrated probe confirmed that the excessive gingival display was stably maintained at 1.5 mm. A harmonious dentolabial relationship and enhanced facial esthetics were maintained, indicating highly satisfactory medium‐term outcomes (Figure 4A–D).

## 4. Patient Perspective

The patient was satisfied with the final esthetic outcome. She reported an improvement in her confidence during social interactions and noted that this minimally invasive procedure yielded significant benefits with only a brief recovery period.

## 5. Discussion

The etiology of excessive gingival display is highly heterogeneous, and an accurate differential diagnosis is a fundamental prerequisite for ensuring the efficacy of clinical interventions. Common etiologies encompass VME, APE, a short upper lip, and hyperactivity of the upper lip elevator muscles [2]. In the present case, the patient exhibited a harmonious proportion of the lower facial third, which effectively ruled out severe skeletal anomalies. Furthermore, the normal clinical crown length (10 mm) combined with the appropriate position of the free gingival margin excluded the possibility of APE, thereby averting an unnecessary crown lengthening procedure. Dynamic smile analysis confirmed that the hypercontraction of the LLSAN and LLS muscles was the direct cause of the gingival exposure. Ultimately, a definitive diagnosis of muscular hyperactive excessive gingival display was established.

For the management of the gummy smile presented in this case, two primary therapeutic modalities are currently available in clinical practice. Local injection of botulinum toxin type A (BTX‐A) represents a common nonsurgical approach [11, 12]. However, in the present case, neurotoxin therapy was deliberately omitted, because the neuromuscular blockade effect induced by BTX‐A injections is transient, typically lasting only 3–6 months. Consequently, patients are subjected to the temporal and financial burdens associated with repeated injections [13].

In contrast, LRS surgically modifies the depth of the maxillary vestibule, creating a structural and mechanical restriction against upper lip elevation, thereby providing a more durable esthetic improvement [13]. The present case utilized this classic surgical technique, which not only effectively mitigates the risk of postoperative midline deviation of the upper lip but also perfectly aligns with the patient′s strong desire for a rapid and relatively long‐lasting esthetic enhancement.

Although the short‐term efficacy of LRS is well established, postoperative relapse remains a primary focal point of concern. Systematic reviews indicate that, owing to the continuous traction exerted by mastication and facial expressions, coupled with tissue memory effects, the partial relapse rate within the first year following LRS is approximately 8%–20% [14]. The primary mechanism of postoperative relapse is mechanically driven by the continuous, high‐power contraction forces exerted by the hyperactive elevator muscles of the upper lip. Following the surgical shortening of the vestibule via mucosal excision, these strong dynamic muscle pulls generate constant, excessive tension on the sutured wound interface. Over time, this mechanical stress leads to the degranulation and stretching of the newly established vestibular attachment, causing the upper lip to revert to its preoperative hypermobile state and resulting in a complete regain of gingival exposure. Consequently, conventional mucosal excision alone is biomechanically inadequate to permanently resist these muscular forces [8]. To counteract the high early muscle tension and mitigate the risk of relapse, the implementation of meticulous suturing techniques is clinically imperative. In the present case, PTFE was utilized, supplemented by rigorous postoperative instructions, with the objective of providing a stable and immobile environment for initial tissue healing. In recent years, some scholars have advocated for the adjunctive preoperative injection of botulinum toxin prior to LRS to secure more stable long‐term outcomes, suggesting that these two therapeutic modalities can be complementary in specific clinical scenarios [5]. To counteract continuous muscular tension and mitigate the high relapse rates, recent literature has proposed several surgical modifications, though their long‐term efficacy remains limited.

### 5.1. Dual‐Layered Suturing Technique

Conventional LRS relies solely on approximating the superficial mucosal edges, which often fails under muscle pull. To address this, a dual‐layered suturing modification was introduced to provide internal fixation by suturing the underlying connective tissue bed prior to external mucosal closure. Although standard LRS may exhibit complete relapse as early as 3 months postoperatively, the dual‐layered approach can temporarily delay the onset of relapse, maintaining initial surgical results for the first 3 months. However, clinical evidence demonstrates that this internal stabilization is ultimately insufficient, as complete relapse is still universally observed by 6 months [9].

### 5.2. Muscle Scarring‐Assisted LRS

Another recent modification involves the intentional transection of muscle fibers combined with rigorous scraping of the underlying wound bed to expose bare bone during the LRS procedure. The biological rationale is to eliminate direct muscle contraction and utilize the resulting dense fibrotic scar tissue to mechanically anchor the lip. Although this aggressive scarring technique successfully delays muscular relapse up to 6 months postoperatively, longitudinal follow‐ups reveal that it fails to provide permanent results, with complete relapse inevitably occurring by 9 months [10]. The present case relied predominantly on the mechanical restriction generated by tissue excision and wound closure, yielding a satisfactory short‐term esthetic outcome; however, a primary limitation of the present study is that it is a single‐case report lacking statistical data from a larger sample size. Therefore, future long‐term prospective clinical trials with larger patient cohorts remain warranted to further refine surgical protocols and systematically evaluate its long‐term esthetic stability.

## 6. Conclusion

In conclusion, provided that strict indications are met and skeletal as well as dental morphological anomalies are definitively excluded, LRS serves as a safe, minimally invasive, and highly predictable interventional strategy for the management of muscular hyperactive gummy smile. Nevertheless, future large‐sample, medium‐term prospective clinical trials remain warranted to further refine surgical protocols and systematically evaluate its long‐term esthetic stability.

## Author Contributions

**Meiya Suo**: investigation, writing—original draft, visualization. **Yu Huan**: data curation, writing—review and editing. **Chenghong Cao**: resources, investigation, validation. **Shuya Zhang**: resources, writing—review and editing, validation. **Lan A. and Wenzhou Xu**: conceptualization, methodology, supervision, project administration, writing—review and editing, funding acquisition.

## Funding

This work was supported by the Natural Science Foundation of Jilin Province (YDZJ202601ZYTS421).

## Disclosure

All authors have read and approved the final version of this manuscript. Each author has contributed significantly to the work and agrees to be personally accountable for their own contributions.

## Ethics Statement

Ethical approval was not required for this single case report. Written informed consent for treatment and publication was obtained from the patient.

## Consent

Written informed consent was obtained from the patient to publish this report, including clinical photographs, in accordance with the journal′s patient consent policy.

## Conflicts of Interest

The authors declare no conflicts of interest.

## Data Availability

The data that support the findings of this study are available from the corresponding authors upon reasonable request.
